# *Falcaria vulgaris* L. hydroalcoholic extract protects against harmful effects of mercuric chloride on the rat kidney

**DOI:** 10.22038/AJP.2023.21872

**Published:** 2023

**Authors:** Ali Ghanbari, Cyrus Jalili, Amir Abdolmaleki, Mohsen Zhaleh, Armin Zarinkhat, Nasim Akhshi

**Affiliations:** 1 *Department of Anatomical Sciences, Kermanshah University of Medical Sciences, School of Medicine, Kermanshah, Iran*; 2 *Student Research Committee, Kermanshah University of Medical Sciences, Kermanshah, Iran*

**Keywords:** Antioxidants, Kidney, Mercuric chloride, Nitric oxide, Oxidative stress

## Abstract

**Objective:**

Mercuric chloride (Merc; HgCl_2_) is toxic to humans and animals and contributes to environmental pollution, which usually results in nerve and systemic harm to different organs. *Falcaria vulgaris* (FV) is a medicinal plant rich in antioxidants. This research aimed to assess the FV hydroalcoholic extract effects on kidney toxicity induced by Merc.

**Materials and Methods:**

Forty-eight male rats were divided into eight groups: the control group: received saline; the Merc group: received 0.5 ml/day of 0.5 ppm aqueous Merc; FV1, 2, and 3 groups: received 50, 100, 150 mg/kg FV, respectively; and Merc + FV1, 2, and 3 groups: received Merc and FV at three doses. The administration period was 14-days. Subsequently, kidneys and sera were cumulated from each group for the analysis. Samples were analyzed via hematoxylin-eosin staining and biochemical tests.

**Results:**

The rats that received Merc displayed significant decrement in the kidney index, the diameter of renal corpuscles, total antioxidant capacity levels, superoxide dismutase activity (all, p<0.01), and 150 mg/kg FV mitigated these outcomes (all, p<0.05). Urea, creatinine, nitric oxide, and the level of apoptosis revealed a significant increment in the kidney of the rats that received Merc (all, p<0.01), and 150 mg/kg FV decreased these results. Furthermore, FV ameliorated histological changes induced by Merc (all, p<0.05).

**Conclusion:**

The FV hydroalcoholic extract protects the kidneys against Merc-induced nephrotoxicity. Antioxidant and anti-apoptotic FV hydroalcoholic extract properties were involved in this healing effect.

## Introduction

Mercury (Hg; one of the toxic heavy metals) due to industrial pollution and its deposition in ecosystems is ubiquitous in the environment and has serious risks to human health. Mercuric chloride (Merc; Hgcl_2_; an inorganic mercury salt) is one of the industrial environmental pollutants that enter the body through respiration, ingestion, skin absorption, and eye contact. It causes damage to the brain, liver, respiratory system, skin, eyes, nervous system, and kidneys (Dash and Das, 2012; Cappelletti et al., 2019). It is believed that Merc toxicity in various tissues is an outcome of reactive oxygen species (ROS) making, resulting in the promotion of inflammatory cytokines and production and increase in nitric oxide (NO) (Cappelletti et al., 2019; Ahmad and Mahmood, 2019). The kidneys are one of the tissues which can damage by Merc poisoning via ROS production and induction of oxidative stress (Yadav et al., 2019; Jalili et al., 2020a). Although there is no efficient method to eschew the toxins' entry into the body, today, natural compounds have enticed the consideration of therapists and researchers to help suffering persons. Previous investigations illustrated the positive role of plant antioxidants on the renal injury caused by methotrexate, nicotine, and Merc-induced damage (Jalili and Jalili, 2018; Yadav et al., 2019; Francis et al., 2020; Jalili et al., 2020a, b).

**Figure 1:**
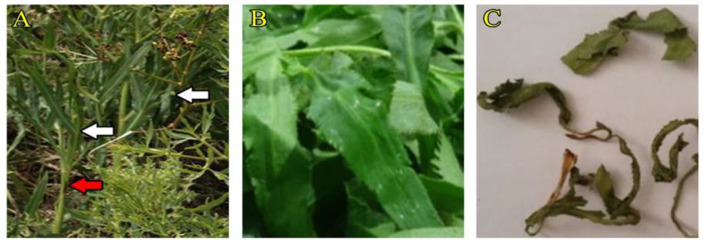
*Falcario vulgaris* L. plant, (A) leaf and stem shown by white and red arrows, respectively, (B) Fresh leaves, (C) Dry leaves (Choobkar et al., 2017).

*Falcaria vulgaris* (FV; Locally named *Ghazzyaghi* / *Poghazeh*) is a medicinal plant is a member of the Apiaceae (Umbelliferae) family, growing in the west and southwest of Iran as an annual wild plant (Figure 1) (Eftekharinasab et al., 2012). In addition to being consumed as a vegetable, this plant is used for healing skin, wound healing, ulcers and peptic ulcers, heart and hematological parameters, and fatty liver disease in folk medicine (Choobkar et al., 2015, 2017; Goorani et al., 2019). In traditional medicine, FV is applied as an antibacterial, antifungal, bleeding inhibitor, and anti-inflammatory treatment (Zangeneh, 2019; Zangeneh et al., 2019). According to the existing studies, the FV extract had antioxidant effects on renal injury in animal models (Zangeneh et al., 2018; jalili et al., 2019a, b). 

Based on the mentioned information, in the current attempt, we studied the potential protective efficacy of FV against Merc-induced nephrotoxicity in male Wistar rats.

## Materials and Methods

**Chemicals**

Merc (Mercury II chloride; HgCl_2_), purity≥99.5% was purchased from Sigma-Aldrich (St Louis, MO, USA). Xylazine was obtained from Alfasan co (Netherlands). Kits from Pars Azmoon, Iran were used to evaluating the serum creatinine and blood urea nitrogen levels. NO level was measured by Griess Reagent System, Promega (Griess reagent). Superoxide Dismutase (SOD) activity in the renal tissue was assayed via Zell Bio kit (GmbH) and spectrophotometric devices. A kit (Cat No: TAC-96A) ZellBioGmbH-Germany was used to estimate the total antioxidant capacity (TAC). Cell death detection was evaluated via ELISA-kit (Cat.No.11544675001; Roche, Germany).

**Preparation of hydroalcoholic extract of FV**

The FV plants were obtained from a local store and approved by botanist Dr. Seyed Mohammad Maassoumi at the herbarium of Razi University, Kermanshah (Code number: RAZ-FPH 221). After removing the impurities, the leaves and stems of the plant were placed in the shade for five days to dry and then powdered. One hundred grams of this powder was combined with 70% ethanol (1:4 ratios) and kept in a warm water bath at 35°C for 48 hr. 

Afterward, it was stored in dark conditions. Next, we used Buchner funnels filter paper, a vacuum pump, and a rotary device to obtain the concentrated extract. Finally, the extract was dissolved in distilled water.

**Animals and experimental protocol**

Forty-eight male Wistar rats (220-250 g) were bought from the Razi Institute of Iran. One week before the start of the experiment, standard cages were used for animal care at 22±2°C temperature and 12-hour light-dark periods. Rats had free access to water and food. Tests were done according to the Helsinki guidelines as well as the Ethics Committee of Kermanshah University of Medical Sciences (IR.KUMS.REC.1398.088).

We randomly divided the 48 rats into eight groups (six animals in each group): Group 1: Control, each animal in this group received 0.9% saline intraperitoneally (I.P), once a day for 14 days; Group 2: Merc, the rats were exposed to a dose of Merc (0.5 mL/day of 0.5 ppm aqueous) dissolved in drinking water once a day for 14 days (Oral); Groups 3-5: FV1, 2, and 3; rats were injected I.P with FV at doses of 50, 100, and 150 mg/kg, respectively once a day for 14 days; and Groups 6-8: Merc+FV1, 2, and 3; the animals received Merc (0.5 ml/day of 0.5 ppm aqueous) (oral) and FV at doses of 50, 100, and 150 mg/kg once a day for 14 days (I.P). The doses of Merc and FV were selected based on preliminary studies (Jalili et al., 2019b; Jalili et al., 2020a). After the last treatment, animals were anesthetized by one I.P injection of ketamine and xylazine at the doses of 100 mg/kg and 10 mg/kg, respectively. Then the kidneys were removed and weighed by digital scales. In the next step, kidney tissue was prepared for macroscopic studies and nitric oxide (NO), SOD, TAC, and apoptosis evaluations. The blood was taken from the hearts and gradually added to the test tubes until the clot formation was maintained at lab temperature. Then serum was separated by centrifugation (1000 g) for 15 min. Blood serums were collected by centrifuging the samples separately and stored at −20°C until blood serum urea nitrogen and creatinine analysis.

**Kidney index evaluation**

The Renal index (mg/g) was estimated using this formula (Ding et al., 2018): Kidney index = Kidney weight (mg)/Body weight (g).

**Histopathological assessments**

The renal tissue was stabilized in buffered formalin (10%) for 24 hr. 

Afterward, a longitudinal incision was used to split each kidney into two sections from the middle. Paraffinized tissue blocks were provided for partitioning at 5-7 μm thickness. 5 microscopic sections of each sample were randomly selected and followed by hematoxylin-eosin (H&E) staining. The slices were studied by a histologist and pathologist using light microscopy (Olympus Optical Co. LTD, Tokyo, Japan). 

For estimation of the diameter of renal corpuscles five fields (25 fields/animals) of 400× and 100× magnifications were captured by a camera connected to the microscope. The field's selection was done via a zigzag form monitoring the sections to achieve a randomized inclusion. Then, at least 150 captured pictures (400×) of the round or nearly round renal corpuscles by a blind observer were selected, and a specialized software package (AE-3; Motic S.L.U., Barcelona, Catalonia, Spain) was used to estimate this parameter. 

**Evaluation of serum urea nitrogen and creatinine**

Serum urea concentration was determined using a commercial kit (Pars Azmoon Co., Tehran, Iran), according to the manufacturer's instructions. Serum creatinine was measured by Jaffe's method, using a commercial Kit (Pars Azmoon Co., Tehran, Iran)**.**

**NO level of the kidney tissue measurement**

Renal tissue NO was assessed by Griess reaction using zinc sulfate powder for deleting proteins from tissue samples. For this aim, 6 mg of zinc sulfate powder was blended with 400 ml of micro tubes of samples. Then, they were homogenized by a vortex for one minute. Afterward, the samples were centrifuged for 10 min at 12,000 revolutions per minute (rpm) at 4 °C. In the next step, an upper solution (supernatant) was utilized to evaluate NO (NOx). In summary, a 50 ml sample was added with 100 ml of Promega (Griess reagent) and the reaction mixture was incubated for about 30 min at room temperature, being preserved from light. The optical density was estimated at 450 nm in a microplate reader in accordance with the reagent manufacturer's instructions.

**Evaluation of the SOD activity**

The reaction between 3-(4,5-dimethylthiazol-2-yl)-2,5 diphenyltetrazolium bromide (MTT) and anion superoxide (made from Pyrogallol) is blocked via the SOD enzyme. In the following, the other steps were done base on the manufacturers' instructions. ELISA at 570 nm was utilized to read the light absorption of this compound. The percentage of inhibition caused by the SOD enzyme was obtained using the kit manufacturer's instructions.

**Estimation of the kidney tissue TAC level**

For measuring the renal tissue TAC, a ZellBioGmbH-Germany kit (Cat No: TAC-96A) was used. The kit contents include a reagent ready to use, dye powder, buffer X 100, reaction suspension solution, standard, and a microplate of 96 wells. The TAC level was assayed by comparing the amount of antioxidants present in one gram of homogenized renal tissue and ascorbic acid was used as the standard (The kit's sensitivity = 0.1 mM). In the end, absorbance was determined using a microplate reader (Model 550, BioRad, Segrate, Milan, Italy) at 490 nm.

**The apoptosis level**

Apoptosis level in the renal tissues was assayed by a cell death detection ELISA kit (Cat.No.11544675001; Roche, Germany). Briefly, 1 mg of the kidney tissue was mixed with 100 µl of lysis buffer and vortexed. The specimens were centrifuged at 1000 g for 10 min and washed with phosphate buffered saline (PBS) in triplicate. The cells suspended in 20 µl of PBS were moved to a 96-well plate filled with 80 µl of immunoreagent. The wells were lidded by a foil for 2 hr at 20°C under vibration (300 rpm). After washing the samples, the absorbance of the samples at a wavelength of 490 nm was read.

**Statistical analysis**

Statistical analysis was done by SPSS (version 16), and the comparisons were performed using one-way ANOVA followed by Tukey's post hoc analysis. The outcomes are demonstrated as mean±SEM (standard error of means). A p<0.05 was assumed statistically significant.

## Results

**Effects of FV and Merc on the kidney index of rat**s

**Figure 2:**
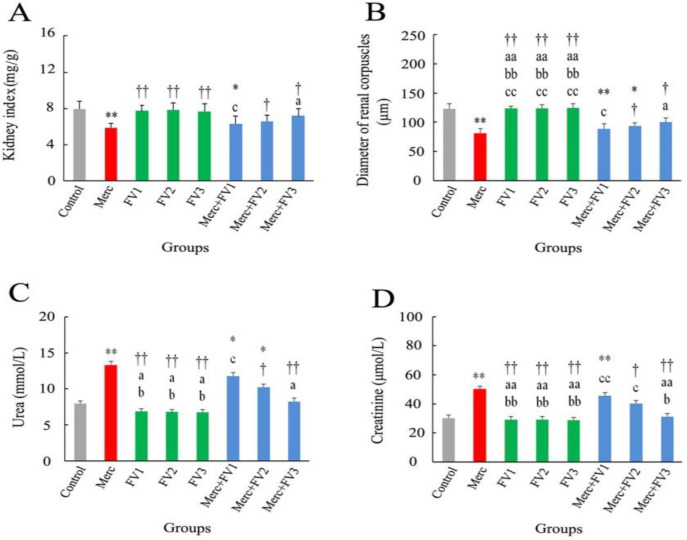
The effect of mercuric chloride (Merc) and *Falcaria vulgaris* L. (FV 1, 2, and 3) on the (A) kidney index, (B) diameter of renal corpuscles, (C) urea, and (D) creatinine. Outcomes are presented as mean±SEM; **p<0.01 and *p<0.05 relative to the Control; ^††^p<00.1 and ^†^p<0.05 compared to the Merc group; ^aa^p<0.01 and ^a^p<0.05 in comparison with the Merc + FV1 group; ^bb^p<0.01 and ^b^p<0.05 versus Merc + FV2 group; ^cc^p<0.01 and ^c^p<0.05 compared to the Merc + FV*3 *group. Merc: Mercuric chloride; FV 1, 2, and 3: *Falcaria vulgaris* at doses 50, 100, 150 mg/kg, respectively.

Evaluation of kidney index displayed a considerable decrement in the Merc (p<0.01) and Merc + FV1 (p<0.05) groups in comparison with the control group. This parameter showed no significant difference for intra-group comparison of the FV1, FV2, and FV3 groups (all, p>0.05). Relative to the Merc group, the kidney index revealed a significant increment in the FV1, FV2, FV3, Merc + FV2, and Merc + FV3 groups (all, p<0.05). Based on Merc + FV intra-groups comparison, the kidney index in the Merc + FV1 group was significantly lower than in the Merc + FV3 group (p<0.05) (Figure 2A).

**Effects of FV and Merc on the kidney histology**

**Figure 3:**
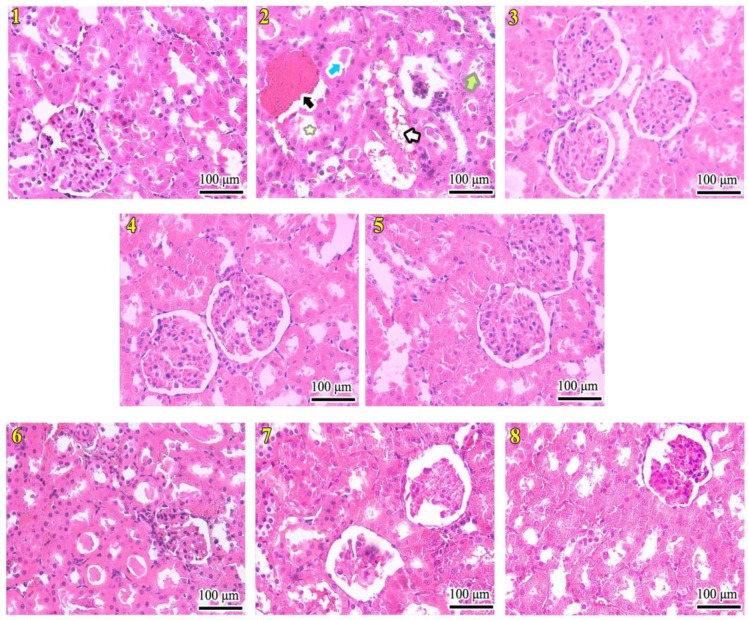
Mercuric chloride (Merc) and *Falcaria vulgaris* L. (FV) effects on the kidney morphology in rats. The numbers in boxes indicate the length of the marked lines in centimeters. Scale bar = 100 μm. (1) Received normal saline (Control). (2) Received mercuric chloride in a dose of 0.5 ml/day. (3-5) Received *Falcaria vulgaris* at 50, 100, and 150 mg/kg doses, respectively. (6-8) Received mercuric chloride in a dose of 0.5 ml/day and *Falcaria vulgaris* at 50, 100, and 150 mg/kg doses, respectively. Green, black, blue and white arrows point to the tubular cell detachment, the vascular congestion, intra-tubular proteinaceous casts, and intra-cellular vacuolization, respectively. The yellow asterisk refers to the tubular dilatation (400× magnification; H & E staining).

As offered in Figure 3 and Table 1, there was an increase in qualitative histological parameters in the Merc, Merc + FV1, and Merc + FV2 groups (all, p<0.01). In the Merc + FV3 group, histological differences were lower than in the control group (p<0.05). In addition, the diameter of renal corpuscles displayed a significant reduction in the Merc, Merc + FV 1 (all, p<0.01), and Merc + FV2 (p<0.05) groups than the control. FV1, FV2, and FV3 intra-groups comparison revealed no difference in renal corpuscle diameter characteristics (all, p>0.05). This parameter showed a significant increment in the FV1, FV2, FV3, Merc + FV2, and Merc + FV3 groups rather than the Merc group (all, p<0.05). Merc + FV1, Merc + FV2, and Merc + FV3 intra-groups comparison revealed that the Merc + FV1 group was significantly lower than the Merc + FV3 group in renal corpuscle diameter characteristics (p<0.05) (Figure 2B).

**Table 1:** Qualitative histology changes in kidney tissue of rats following treatment with mercuric chloride (Merc), *Falcaria vulgaris* L. (FV) or both.

**Groups**								
**Histopathology Indices**	Control	Merc	FV1	FV2	FV3	Merc+FV1	Merc+FV2	Merc+FV3
Tubular cell detachment	0	III	0	0	0	III	III	0
Vascular congestion	0	VI	0	0	0	II	I	0
Intra-tubular proteinaceous casts	0	VI	0	0	0	IV	II	0
Intra-cellular vacuolization	0	II	0	0	0	III	II	I
Tubular dilatation	0	VIII	0	0	0	V	III	I
Total	0	XXV^**^	0 ^††^^,aa^	0 ^††^^,aa^	0 ^††^^,aa^	XVII^**, a^	XI^**, ^^†, ^^aa^	II^*, ^^††, ^^aa^

Merc, Mercuric chloride, FV, *Falcaria vulgaris* L.**p<0.01 and *p<0.05 relative to the Control; ^††^p<00.1 and ^†^p<0.05 relative to the Merc group; ^aa^p<0.01 and ^a^p<0.05 in comparison with the Merc + FV1 group

**Effects of FV and Merc on urea and creatinine** **serum levels **

There was a significant increase in urea serum level (Figure 2C) and creatinine (Figure 2D) level in the Merc group compared to the control (all, p<0.01). Also, the Merc + FV1 group showed a considerable increase for these two parameters compared to the control group (all, p<0.05) (Figures 2C-D). Serum levels of urea revealed a significant increment in the Merc + FV2 group compared to the control group (p<0.05) (Figure 2C). The urea and creatinine serum levels alterations in the intra-group comparison of FV alone recipients were not statistically significant (all, p>0.05). FV administration in the FV1, FV2, FV3, Merc + FV2, and Merc + FV3 groups significantly decreased both parameters than the Merc group (all, p<0.05). Intergroup comparison of Merc + FV groups showed that compared to the Merc + FV1 group, a significant reduction was observed for these two parameters in the Merc + FV3 group (all, p<0.05). Also, serum levels of creatinine showed a remarkable decrease in the Merc + FV3 group compared to the Merc + FV2 group. (p<0.05) (Figures 2C-D).

**Effects of FV and Merc on NO level, SOD activity, and TAC level in renal tissue of rat**s

**Figure 4:**
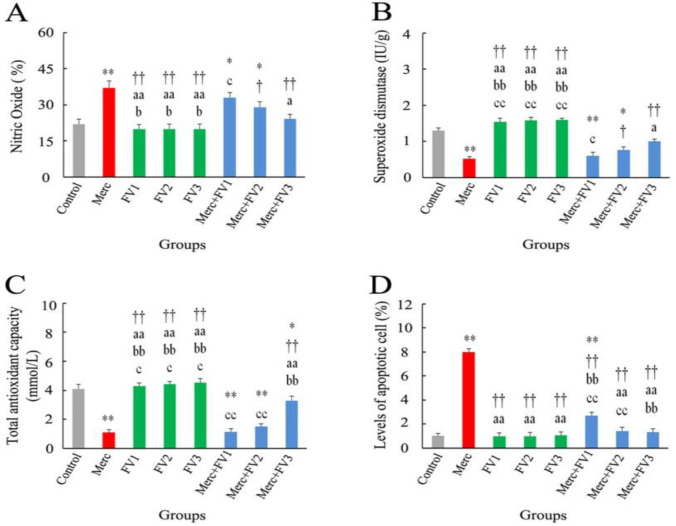
The effect of mercuric chloride (Merc) and *Falcaria vulgaris* L. (FV 1, 2, and 3) on the (A) Nitric oxide (NO), (B) Superoxide dismutase (SOD), (C) Total antioxidant capacity (TAC), and (D) Levels of apoptotic cell. Outcomes are presented as mean±SEM; **p<0.01 and *p<0.05 relative to the Control; ^††^p<00.1 and ^†^p<0.05 compared to the Merc group; ^aa^p<0.01 and ^a^p<0.05 in comparison with the Merc + FV1 group; ^bb^p<0.01 and ^b^p<0.05 versus Merc + FV2 group; ^cc^p<0.01 and ^c^p<0.05 compared to the Merc + FV*3 *group. Merc: Mercuric chloride; FV 1, 2, and 3: *Falcaria vulgaris* at doses 50, 100, 150 mg/kg, respectively.

There was a significant increase in renal tissue NO levels in the Merc (p<0.01), Merc + FV1 (p<0.05), and 2 (p<0.05) groups compared to the control group. The alterations of this parameter in the intra-group comparison of FV alone recipients were not statistically significant (all, p>0.05). FV administration in the FV1, FV2, FV3, Merc + FV2, and Merc + FV3 groups significantly decreased renal tissue NO levels than the Merc group (all, p<0.05). In the Merc + FV groups intra-group comparison, this parameter in the Merc + FV1 group was significantly higher relative to the Merc + FV3 group (p<0.05) (Figure 4A). The renal tissue SOD and TAC levels showed a significant decrement in the Merc, Merc + FV1 (all, p<0.01), and Merc + FV2 (p<0.05) groups relative to the control group. Moreover, the Merc + FV3 group showed a significant decrease in TAC level compared to the control group (p<0.05). These two parameters did not indicate significant differences in the FV1, 2, and 3 intra-groups comparison (all, p>0.05).

Compared to the Merc group, there were remarkable increases in the renal SOD and TAC levels in the FV 1,2,3 and Merc + FV3 groups. Also, renal SOD levels showed a considerable enhancement in the Merc + FV2 group in comparison with the Merc group (p<0.05). In the Merc + FV intra-group comparison, the renal SOD and TAC in the Merc + FV1 group were remarkably lower than in the Merc + FV3 group (all, p<0.05) (Figure 4B-C).

**Effects of FV and Merc on the number of apoptotic cells**

According to Figure 4D, apoptotic cells count revealed a remarkable enhancement in the Merc and Merc + FV1 groups compared to the Control group (all, p<0.01). Comparing intra-groups treatment with alone FV showed no significant effect on apoptotic cells (all, p>0.05). Compared with the Merc group, six FV-treated groups demonstrated a significant decrease in apoptotic cell counts (all, p<0.01). In the Merc + FV groups comparison, this parameter showed a significant reduction when the FV concentration was increased (all, p<0.01).

## Discussion

Merc poisoning is one of the factors that increase ROS production in cells. It induces organ injury by incrementing the free radicals' production and oxidative stress (Cappelletti et al., 2019; Hosseini et al., 2018). Since oxidative stress is involved in Merc poisoning, it has been suggested that antioxidants could help in its treatment. Therefore, researchers have focused on finding natural antioxidants of plant origin. FV is a medical plant with antioxidant activity (Zangeneh et al., 2018; Jalili et al., 2019a, b). Thus, in this attempt, we studied the effect of FV hydroalcoholic extract on Merc-induced toxicity in renal tissue of male rats. Generally, our results demonstrated that FV administration protected the studied rats against Merc-induced nephrotoxicity by enhancing antioxidant defense status.

In this study, the kidney index decrement in the Merc alone receiving group was seen to occur due to their weight loss which is described via kidney parenchyma atrophy and decline of the renal corpuscle size as a result of apoptosis induction. Similar to our results, Abdel-Moneim and colleagues have reported a reduced kidney index of rats following lead acetate administration (Abdel-Moneim et al., 2011). On the other hand, the FV extract effectively restored kidney and body weight loss in rats receiving Merc. 

After administrating FV in Merc-induced groups, the diameter of renal corpuscles elevated. In line with our results, a study revealed that the renal corpuscles diameter decreased in Merc-induced mice (Jalili et al., 2020a). The histological estimation affirmed the ameliorative efficacy of FV on Merc-induced nephrotoxicity. It seemed that Merc via oxidative stress induction caused renal tissue injury. Other investigations have reported Merc-induced histological damages (Gao et al., 2016; Jalili et al., 2020a). Merc via enhancement of ROS production could activate nuclear factor kappa B and lead to the over secretion of proinflammatory cytokines. It seemed that inflammation was an important mechanism involved in Merc-induced nephrotoxicity. Previous researchers reported an increment in the concentration of inflammation factors in animal renal tissue after Merc treatment (Li et al., 2018; Almeer et al., 2019).

Outcomes of the current study show that Merc-induced kidney injury is evidenced by elevation in the serum urea and creatinine, as revealed in another research (Gado and Aldahmash, 2013; Gao et al., 2016; Yadav et al., 2019; Francis et al., 2020; Jalili et al., 2020a). The elevated value of urea and creatinine is due to oxidative stress-induced cellular injury and reduced glomerular filtration rate which shows the loss of kidney function (Rule et al., 2004; Silva and Coutinho, 2010). The free radicals produced by oxidative stress change the enzymatic activity via assaulting biological molecules like DNA, proteins, lipids, and proteins, which finally cause cell injury or death (Silva and Coutinho, 2010). In contrast, FV administration significantly restored the changed levels of urea and creatinine that might be due to the repair of tissue injury mediated by its antioxidants' effects. It seems that polyphenol hydroxyl groups existing in FV arrested the free radicals (Monfared et al., 2012). In conformance to these results, previous research showed that FV hydroalcoholic extract treatment reduced urea and creatinine levels and improved kidney tissue and kidney function mediated by reducing renal biochemical markers levels (Jalili et al., 2019b). 

Based on our results, the FV hydroalcoholic extract effectively reduced renal tissue NO level and increased tissue levels of SOD and TAC in rats receiving Merc. Mitochondrial dysfunction in most body tissues increases the production of free radicals such as NO and ultimately induces tissue damage (Carpino et al., 2017). NO has a role in acute kidney failure because its free-radical nature might participate in tubular harm (Christo et al., 2011). As reported previously, Merc intoxication enhances the creation of pro-inflammatory factors and NO in mice (Piacenza et al., 2009; Omanwar et al., 2013). Also, the findings of Jalili and colleagues confirmed that Merc treatment increased NO levels in renal tissue of mice while reducing the production of TAC and SOD activity in this tissue, which is consistent with the results of the present study (Jalili et al., 2020a). According to our results, FV can reduce the levels of free radicals causing reduced levels of NO and enhancing the levels of antioxidant activity. FV appears to exert protective effects against Merc-induced nephrotoxicity via enhancing the kidney antioxidant capacity and inhibiting oxidative stress in this tissue. In line with these results, other studies have shown the antioxidant properties of FV (Rafiey et al., 2017; Zangeneh et al., 2018; Jalili et al., 2019a,b).

Our findings revealed the increment in the renal tissue level of apoptosis following Merc administration which is consistent with previous reports (Pal et al., 2012; Jalili et al., 2020a). Based on the current study, the administration of FV in Merc receiving groups significantly decreased the level of apoptotic cells. It indicated the role of FV in apoptosis inhibition. In line with our results, Hosseini and colleagues have reported that FV has significant cytotoxic effects on SW-872 cells via apoptosis induction (Hosseini et al., 2021). 

Based on the results of the present study, there was no significant difference between the FV alone receiving groups for any investigated parameters. Moreover, there were remarkable differences between the groups that received Merc + FV at 50 mg/kg and Merc + FV at 150 mg/kg for all the investigated parameters. The data showed there are no differences between groups of Merc + FV at 50 mg/kg and 100 mg/kg. Therefore, we suggested that a higher dose of FV (150 mg/kg) was more impressive effects on the Merc nephrotoxicity.

According to the outcomes of this study, Merc triggers oxidative stress and the production of free radicals in kidney tissue. In contrast, the FV plant's hydroalcoholic extract reduced Merc poisoning's effects on kidney tissue. This reduction in toxicity is justifiable due to the saponin, flavonoid, and antioxidant compounds in FV extract that decline the NO level, increase the SOD activity, and down-regulation of apoptosis. Thus, our results help to understand how environmental doses of Merc interact in their biological effects on kidney tissue. Research on the effective mechanism for applying this protective effect can help develop appropriate treatments with the minimum side effects for humans and animals exposed to heavy metals.

## Conflicts of interest

The authors have declared that there is no conflict of interest.
